# Effect of Compressive Prestrain on the Anti-Pressure and Anti-Wear Performance of Monolayer MoS_2_: A Molecular Dynamics Study

**DOI:** 10.3390/nano10020275

**Published:** 2020-02-06

**Authors:** Ning Kong, Boyu Wei, Yuan Zhuang, Jie Zhang, Hongbo Li, Bo Wang

**Affiliations:** 1 School of Mechanical Engineering, University of Science and Technology Beijing, Beijing 100083, China; 2 Beijing Institute of Spacecraft System Engineering, Beijing 100094, China

**Keywords:** monolayer MoS_2_, compressive prestrain, anti-wear, molecular dynamics

## Abstract

The effects of in-plane prestrain on the anti-pressure and anti-wear performance of monolayer MoS_2_ have been investigated by molecular dynamics simulation. The results show that monolayer MoS_2_ observably improves the load bearing capacity of Pt substrate. The friction reduction effect depends on the deformation degree of monolayer MoS_2_. The anti-pressure performance of monolayer MoS_2_ and Pt substrate is enhanced by around 55.02% when compressive prestrain increases by 4.03% and the anti-wear performance is notably improved as well. The improved capacities for resisting the in-plane tensile and out-of-plane compressive deformation are responsible for the outstanding lubrication mechanism of monolayer MoS_2_. This study provides guidelines for optimizing the anti-pressure and anti-wear performance of MoS_2_ and other two-dimension materials which are subjected to the in-plane prestrain.

## 1. Introduction

Since graphene has been successfully developed by mechanical exfoliation method in 2004 [1], two-dimensional (2D) materials have attracted more and more attentions on this research topic. 2D materials present extremely strong covalent bond interactions in molecular layer, while there is only a relatively weak van der Waals force in the interlayer. The special layered structure endows them with excellent mechanical performance [2,3,4,5,6] and tribological performance [7,8,9,10,11,12]. The discovery and preparation of 2D materials, as represented by graphene and molybdenum disulfide (MoS_2_), provides new directions for the development and improvement of the solid lubricant materials.

Both atomic force microscopy (AFM) experiments [4,13] and molecular dynamics (MD) simulation [14,15] have indicated that MoS_2_ shows an excellent mechanical property, with the effective Young’s modulus of 270 ± 100 GPa and the average breaking strength of about 23 GPa [4]. Macroscopic friction experiments indicate that MoS_2_ possesses an excellent lubrication property [16] even in vacuum [17,18,19], while graphene shows a poor lubrication property in dry conditions [20,21,22]. It indicates that MoS_2_ is an ideal substitute material for graphene in terms of environmental sensitivity. Meanwhile, monolayer MoS_2_ or thin film MoS_2_ is commonly used as a solid lubricant in microelectromechanical systems (MEMS) which presents a prominent lubrication property [7,8,9]. It is applied to avoid excessive thickness of the lubricant layer which may decrease the performance of the micro-device [23,24].

In principle, in order to enable MoS_2_ to show excellent tribological properties, large-area and pristine MoS_2_ films are required. Large area monolayer MoS_2_ could be synthesized by chemical vapor deposition (CVD) [25,26,27,28]. However, monolayer MoS_2_ tends to accumulate straining due to the lattice mismatch between MoS_2_ and substrate [29,30], the difference in thermal expansion coefficient between MoS_2_ and substrate [31], and the coating parameters of CVD [32]. The existence of prestrain is able to change the physical properties of MoS_2_, such as electronic and optical properties [33,34]. The tribological properties of other 2D lubricating materials (such as graphene) could be also affected by their prestrains [35,36,37]. However, the effect of compressive prestrain on the tribological properties of monolayer MoS_2_ is still unclear. An in-depth understanding is essential for the anti-pressure and anti-wear performances of monolayer MoS_2_ with compressive prestrain supported by realistic metallic substrate. It is also responsible for the practical applications of the monolayer MoS_2_ under required extreme contact conditions.

In this work, a many-body reactive empirical bond-order (REBO) potential, an embedded atom method (EAM) potential and Lennard-Jones (LJ) potentials are used to describe the interaction in the simulation model. The atomic morphology of monolayer MoS_2_ was observed after relaxation process. The MD simulation has been applied to investigate the effects of the compressive prestrain on the anti-pressure and anti-wear performance of monolayer MoS_2_. It is quite different from the atomic scale tribological mechanism of an ideal monolayer MoS_2_ with different initial states. The parameters of uniaxial compression have been adjusted in order to preset the compressive prestrain of monolayer MoS_2_. The monolayer MoS_2_ that supported by a platinum substrate with compressive prestrain has been simulated during atomistic nano-indentation and nano-scratch processes. The simulation results confirm that the monolayer MoS_2_ observably improves the load bearing capacity of the Pt substrate. The anti-pressure performance of monolayer MoS_2_ is enhanced by the increase of the in-plane compressive prestrain. The friction reduction of monolayer MoS_2_ depends on the deformation degree of monolayer MoS_2_. The anti-wear performance of monolayer MoS_2_ could be enhanced with the increase of the in-plane compressive prestrain. Based on the results above, the structural deformations of monolayer MoS_2_ during the indentation and sliding processes have been deeply investigated, which may provide guidelines for optimizing the anti-pressure and anti-wear performance of monolayer MoS_2_.

## 2. Materials and Methods

**Figure 1:**
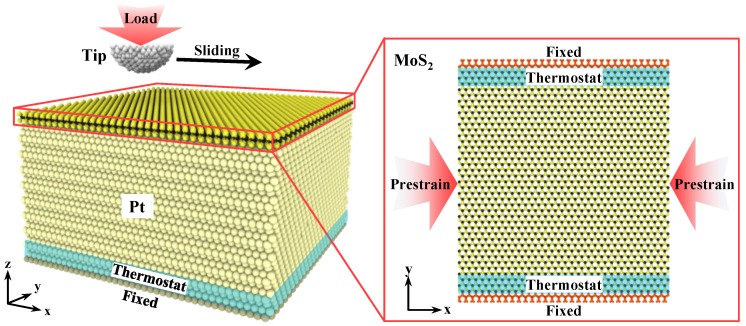
MD simulation model of a diamond tip and MoS_2_/Pt substrate.

The simulation models in this work involved a rigid diamond tip interacted with monolayer MoS_2_. The Pt is chemical stabilization, which is often used as a substrate to support 2D lubricating materials. The monolayer MoS_2_ is covered on a face-centered cubic Pt(111) substrate, as shown in Figure 1. The radius of the tip was 15 Å and the thickness of Pt(111) substrate was 58.907 Å. The prestrain was applied to the monolayer MoS_2_ in *x*-direction by uniaxial compression in order to investigate the effects of compressive prestrain on the anti-pressure and anti-wear performance of monolayer MoS_2_. It is noteworthy that there is a lattice mismatch in the in-plane periodicities of overlayer and substrate when MoS_2_ is synthesized by means of CVD. With mica substrate, the lattice constant of α_(mica)_ is 0.531 nm while the α_(MoS2)_ is 0.315 nm. The α_(mica)_ is approximately 1.7-times greater than the α_(MoS2)_ with a mismatch of −2.7% (the negative sign means MoS_2_ lattice is compressed). This indicates that the rotation of MoS_2_ lattice by about 30° would result in a commensurate structure with mica, which may also induce an epitaxial growth of MoS_2_ on mica substrate [29]. Similarly, a maximal mismatch of −3.6% could be estimated when MoS_2_ is synthesized on the Pt substrate. On the consideration of lattice mismatch, thermal expansion coefficient between MoS_2_ and substrate, coating parameters and the periodic boundary condition, the compressive prestrain conditions are tested up to 4.03%. The lengths of all monolayer MoS_2_ in *x*-direction have been provided in Table 1 and the length of monolayer MoS_2_ in *y*-direction was 115.348 Å. The lateral size of Pt substrate was the same as monolayer MoS_2_. The initial distance between Pt(111) surface and monolayer MoS_2_ was 2.8 Å where the interaction energy between MoS_2_ and Pt(111) surface was at a minimum. In order to simulate indentation and sliding processes, the MoS_2_ atoms at the edge in the *y* direction were anchored in both the *x* and *y* directions, but were able to move freely along the *z* direction to avoid the translational motion of the whole monolayer MoS_2_. The bottom atoms of Pt were fixed to support the MoS_2_/Pt substrate.

In order to study the intra-layer interaction of MoS_2_, a many-body reactive empirical bond-order (REBO) [38] was chosen because of its great ability to simulate the covalently bonded systems of MoS_2_ and dynamically describe the breaking and recombining events of Mo–S bonds [14]. The pairwise REBO parameters used in this work is from relative references [39]. The interaction between Pt atoms in the substrate was described by an embedded atom method (EAM) potential [40]. The van der Waals forces among the diamond tip, monolayer MoS_2_ and Pt substrate were described by C–S, C–Mo, C–Pt, S–Pt, and Mo–Pt Lennard-Jones (LJ) potentials. The parameters were obtained by Lorentz–Berthelot mixing rules [41,42,43,44] and listed in Table 2. During the indentation process, the diamond tip was moved perpendicularly to the surface of the MoS_2_/Pt substrate, while the diamond tip was moved horizontally under different load depths during the sliding process. On the consideration of heat dissipation effect during sliding of the diamond tip, a thermostat scheme is adopted to the model. Sliding speeds must be at a level of m/s to capture sufficient sliding distances to observe multiple periods of stick-slip. On the consideration of thermal activation mechanism, the diamond tip moves at a speed of 20 m/s during the indentation and sliding process. The Langevin thermostat [42] was applied to the specified regions which are adjacent to the fixed atoms of the MoS_2_ and Pt in order to maintain the system temperature at 300 K, as shown in Figure 1. It should be noted that the thermostat is applied only to the atoms away from the contact region to minimize the impact of numerical thermostat on the dynamics of the system [45], as shown in Figure S1. Each simulation was repeated three times in order to confirm the reliability of the results, as shown in Figure S2. All the simulations were conducted by means of the MD simulation package LAMMPS.

## 3. Results and Discussion

### 3.1. Relaxation Process

**Figure 2:**
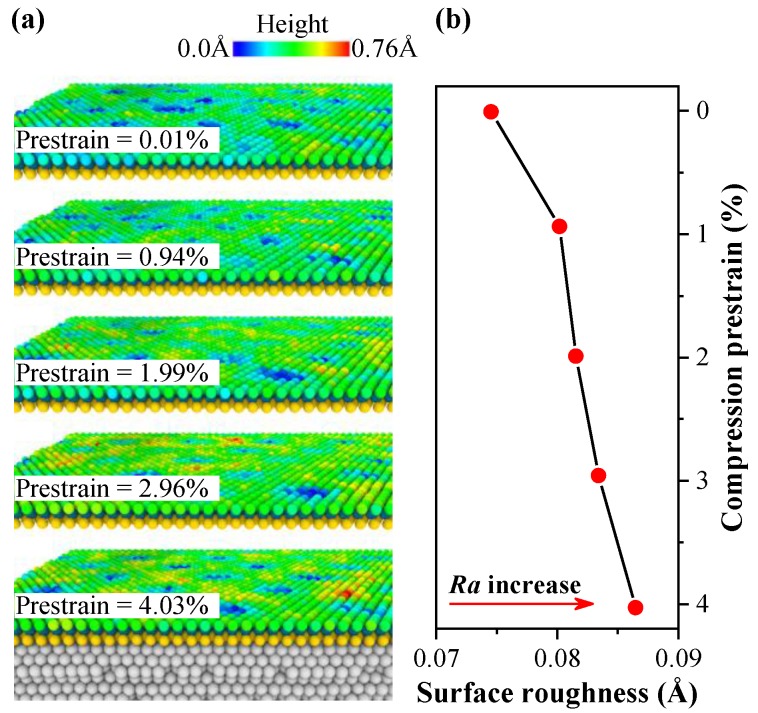
After relaxation process, (**a**) atomic morphology of monolayer MoS_2_ (color symbolizing the atomic heights of S layer on the top). (**b**) Surface roughness of the monolayer MoS_2_ under different compressive prestrain conditions.

The increase of the compressive strain in-plane of 2D materials may enhance the degree of fluctuations and puckering of the material to some extent [37]. The atomic morphology of monolayer MoS_2_ was observed after relaxation process for evaluating the effects of compressive prestrain on the degree of fluctuations of monolayer MoS_2_. The S atoms on the top layer were colored according to the height of the atom position, as shown in Figure 2a. From Figure 2a, the surface morphology of monolayer MoS_2_ with 0.01% prestrain is quite flat, which is consistent with previous study [46]. With the increase of compressive prestrain, the fluctuations of monolayer MoS_2_ increases slightly, but the monolayer MoS_2_ is not appreciably buckled. The surface roughness of monolayer MoS_2_ with different compressive prestrain has been obtained by calculating the arithmetical mean deviation of the surface profile, as shown in Figure 2b. The surface roughness increases slightly with the increase of compressive prestrain. However, it is generally in a lower level.

The bending modulus of monolayer MoS_2_ is 9.61 eV, which is much higher than the monolayer graphene with a bending modulus of 1.4 eV. Monolayer MoS_2_ presents an advantage over graphene on resisting against buckling owing to its bending modulus is about 7 times higher than the graphene [47]. Each monolayer of S atoms contributes 1.75 eV to the bending modulus, which is similar to the bending modulus of monolayer graphene with 1.4 eV. However, the additional pairwise and angular interactions between the Mo and S atoms contribute 5.84 eV to the bending modulus. Compared to the graphene, the higher bending modulus of MoS_2_ is attributed to its layer thickness. Therefore, the three layers of atoms, the bond interaction and angular interaction in MoS_2_ contribute to the bending modulus. The ability on resisting normal instability is enhanced because of the strong covalent bond interaction between atoms in MoS_2_ as well. In addition, the contact surface between monolayer MoS_2_ and Pt substrate is relatively flat, which reduces the deformation of MoS_2_.

### 3.2. Indentation Process

**Figure 3:**
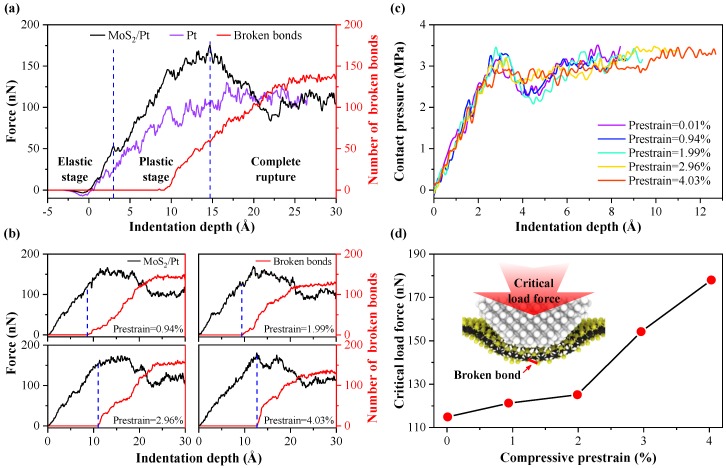
(**a**) The force-depth relations of the MoS_2_/Pt substrate (black curve) and the bare Pt substrate (blue-violet curve) with the indentation process, the number of broken bonds in monolayer MoS_2_ during the indentation process (red curve). (**b**) The force-depth curves (black curve) and the number of broken bonds (red curve) of the MoS_2_/Pt substrate with different compressive prestrains. (**c**) The contact pressure curves between the diamond tip and MoS_2_ before the bonds breaks. (**d**) The critical load forces of the MoS_2_/Pt substrate with different compressive prestrains.

The atomistic nano-indentation process of monolayer MoS_2_ with prestrain of 0.01% supported by a platinum substrate has been investigated before studying the effects of compressive prestrain on the anti-pressure performance of monolayer MoS_2_. The normal force acting on the diamond tip has been recorded for each indentation step (black curve in Figure 3a). An analogous simulation with a bare Pt substrate was performed (blue-violet curve in Figure 3a) in order to assess the protection capability of monolayer MoS_2_. The indentation depth was preset to zero in the position where the attraction force was equal to the repulsion force between the diamond tip and substrate. To evaluate the rupture degree of the monolayer MoS_2_, the number of broken bonds in monolayer MoS_2_ was counted (red curve in Figure 3a). It can be seen from Figure 3a that the normal force for MoS_2_/Pt substrate is always greater than the bare Pt substrate at same indentation depth when the rupture degree of MoS_2_ was slight. While the two force-depth curves almost overlap after MoS_2_ is completely ruptured. The result above is consistent with previous study [48], indicates that monolayer MoS_2_ improves the load bearing capacity of the Pt substrate.

Three stages could be divided for the force-depth relation curve in Figure 3a: elastic stage (the smooth part of force-depth curves, with indentation depth < 3.0 Å for the MoS_2_/Pt substrate, indentation depth < 2.1 Å for the bare Pt substrate), plastic stage and the complete rupture stage of monolayer MoS_2_. At the plastic stage, covered monolayer MoS_2_ delays the beginning point of the plasticity stage for the Pt substrate, which is similar to the tendency of previous study on graphene [49]. Sawtooth shaped steps appear in the force-depth curves because of the dislocation activity inside the Pt substrate. The force-depth curves for the indentation process of the MoS_2_/Pt substrate with different compressive prestrain are shown in Figure 3b. The trends of these curves are similar to the curve in Figure 3a, which also could be divided into three stages. To verify the boundary between the elastic stage and plastic stage, the contact pressure is calculated by the load force and the actual contact area [50] between the diamond tip and MoS_2_, as shown in Figure 3c. It can be seen from the contact pressure curve that the contact stress rises monotonously when the indentation depth is less than 3.0 Å. It conforms to the basic law of elastic deformation.

**Figure 4:**
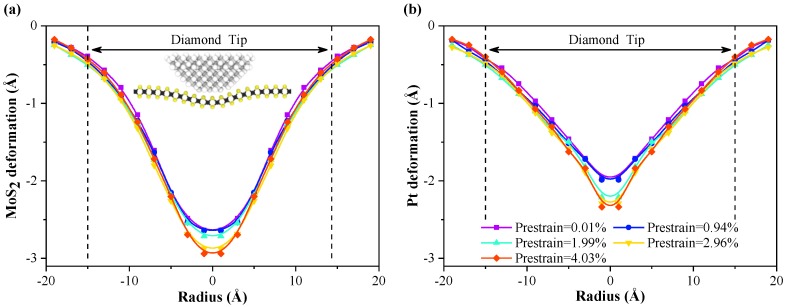
Radially averaged deformation distribution of monolayer MoS_2_ (**a**) and Pt substrate (**b**) at the end of the elastic stage, obtained by averaging the atomic displacement from the center of the contact area at radial intervals of 2.0 Å. To better reflect the deformation of the Pt substrate, only the top three layers of Pt atoms are considered.

In Figure 3b, the smoothness of the force-depth curve decreases with progression through the elastic stage and with increasing compressive prestrain. The boundary between the elastic stage and the plastic stage is not obvious when the compressive prestrain reaches 4.03%. To explain the above phenomenon, the deformations of monolayer MoS_2_ and Pt substrate are calculated at the end of elastic stage, as shown in Figure 4. The deformation is obtained by averaging the atomic displacement along the *z* direction from the center of the contact area at a radial interval of 2.0 Å. The out-of-plane deformation of 2D-material with compressive prestrain is more likely to form due to the decrease of the in-plane tensile stress [51,52]. From Figure 4a, the out-of-plane deformation of monolayer MoS_2_ increases with increasing compressive prestrain at a given indentation depth. A larger deformation of the Pt substrate will occur consequentially, as shown in Figure 4b. The dislocation activity inside the Pt substrate is more pronounced, resulting in a decrease of the smoothness of force-depth curves and a blurring of the boundary between the elastic and plastic stage.

In order to compare the anti-pressure performance of the monolayer MoS_2_ with different compressive prestrain, the critical loading forces have been calculated, as shown in Figure 3d. The critical loading force is defined as the normal force acting on the diamond tip when the broken bonds occur in the monolayer MoS_2_. With compressive prestrain increases from 0.01% to 4.03%, the critical loading depth of monolayer MoS_2_ and Pt substrate increases by 51.19%, from 8.4 Å to 12.7 Å. The critical loading force of monolayer MoS_2_ and Pt substrate increases by 55.02%, from 114.84 nN to 178.03 nN. It can be concluded that the in-plane compressive prestrain improves the anti-pressure performance of the monolayer MoS_2_.

To understand the fracture mechanism in monolayer MoS_2_ with different compressive prestrains, the structural deformations of monolayer MoS_2_ during the indentation process have been investigated. Unlike graphene, which forms a single atomic layer structure, monolayer MoS_2_ consists of three layers of atoms. Therefore, the deformation mechanism of monolayer MoS_2_ is more complicated than that of graphene due to the structural differences. In this work, two main structural deformations during the indentation process were investigated, including in-plane tensile deformation and out-of-plane compression deformation [53]. The in-plane tensile deformation could be represented by the distance changes between the Mo atoms, while the out-of-plane compressive deformation could be represented by the distance changes between two relative S atoms [54]. Besides, the length of Mo–S bonds is used to represent the combined influence of both deformations.

**Figure 5:**
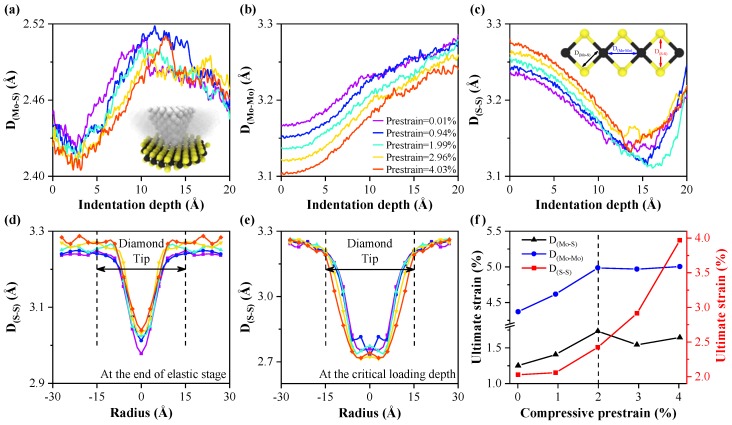
Evolution of D_(Mo–S)_ (**a**) D_(Mo–Mo)_ (**b**) and D_(S–S)_ (**c**) for atoms in the contact area between the diamond tip and MoS_2_ during the indentation process. Radially averaged distance distribution of D_(S–S)_ for MoS_2_ at the end of the elastic stage (**d**) and at the critical loading depth (**e**), which is obtained by averaging the D_(S–S)_ from the center of the contact area at radial intervals of 3.0 Å. (**f**) The ultimate strains of D_(Mo–S)_, D_(Mo–Mo)_, and D_(S–S)_ before the critical loading depth of each model, respectively.

The calculated structural deformations area should be limited to avoid the influence of the surrounding undeformed area of MoS_2_. Therefore, the deformation processes of MoS_2_ are investigated in the contact area [50] between the diamond tip and MoS_2_. The average length of Mo–S bonds (D_(Mo–S)_), the average distance between adjacent Mo atoms (D_(Mo–Mo)_) and between two relative S atoms (D_(S–S)_) during the indentation process are calculated, as shown in Figure 5a–c. The effective values of D_(Mo–S)_, D_(Mo–Mo)_ and D_(S–S)_ are all short than the maximum cutoff radius [39]. It is evident from Figure 5a–c that initial values of D_(Mo–S)_, D_(Mo–Mo)_ and D_(S–S)_ are modulated regularly by compressive prestrain prior to the indentation process. During the indentation process, the D_(Mo–S)_ decreases at the elastic stage. With a further increase of indentation depth, the D_(Mo–S)_ increases continuously until MoS_2_ is completely ruptured. The D_(S–S)_ decreases regularly before the indentation depth reaches the critical loading depth. While the D_(S–S)_ is out of regularity as the indentation depth increases further. The above phenomenon indicates that the variation of the D_(Mo–S)_ and D_(S–S)_ are related closely to the location of the indentation depth, while the D_(Mo–Mo)_ increases continuously with indentation depth during the indentation process. Therefore, the effect of out-of-plane compression deformation on the rupture of MoS_2_ is greater than the in-plane tensile deformation [48].

The radial distribution of D_(S–S)_ has been calculated at various indentation depths in order to better reflect the out-of-plane compression deformation of monolayer MoS_2_. At the end of the elastic stage, as shown in Figure 5d, the compression deformation is more concentrated in the center of the contact area between the diamond tip and MoS_2_. The minimum value of D_(S–S)_ decreases as compressive prestrain increases, while the relative deformation value is similar because of the different initial values of D_(S–S)_. At the critical loading depth of each model, as shown in Figure 5e, the compression deformation of MoS_2_ is more signficant, while the minimum values of D_(S–S)_ remains nearly unchanged with increasing compressive prestrain. There is a critical value of D_(S–S)_ for rupture caused by the out-of-plane compression of MoS_2_, and the critical value of D_(S–S)_ is close to 2.75 Å.

In order to further explain the reasons that the in-plane compressive prestrain is able to enhance the anti-pressure performance of the monolayer MoS_2_, the ultimate strain of D_(Mo–S)_, D_(Mo–Mo)_, and D_(S–S)_ before the critical loading depth of each model are calculated, as shown in Figure 5f. With an increase in the in-plane compressive prestrain of monolayer MoS_2_, the ultimate strain of D_(Mo–Mo)_ increases at first and then remains at a certain level, while the ultimate strain of D_(S–S)_ increases continuously. The in-plane compressive prestrain of monolayer MoS_2_ affects the ultimate strain of the in-plane tensile deformation and the out-of-plane compressive deformation, resulting in the variation in the anti-pressure performance of the monolayer MoS_2_. When the in-plane compressive prestrain is less than 2%, both ultimate strain of D_(Mo–Mo)_ and D_(S–S)_ increases with the increasing compressive prestrain, which improves the capacities of MoS_2_ to resist the in-plane tensile deformation and out-of-plane compressive deformation. However, when the in-plane compressive prestrain is greater than 2%, only the ultimate strain of D_(S–S)_ increases with compressive prestrain. The D_(Mo–Mo)_ and D_(S–S)_ are the main indicators for the structural deformations of monolayer MoS_2_. The in-plane tensile deformation could be represented by the distance changes between the Mo atoms (D_(Mo–Mo)_), while the out-of-plane compressive deformation could be represented by the distance changes between two relative S atoms (D_(S–S)_). The length of Mo–S bonds (D_(Mo–S)_) is used to represent the combined influence of both deformations. The D_(Mo–S)_ is influenced by both D_(Mo–Mo)_ and D_(S–S)_. Therefore, the dependence of the ultimate strain is different for D_(Mo–S)_ and D_(Mo–Mo)_. It mainly improves the capacity of MoS_2_ to resist the in-plane tensile deformation. Therefore, at various in-plane compressive prestrain, the capacities of MoS_2_ to resist the in-plane tensile deformation and out-of-plane compressive deformation can be improved to various degrees, and this enhances the anti-pressure performance of the monolayer MoS_2_.

### 3.3. Sliding Process

**Figure 6:**
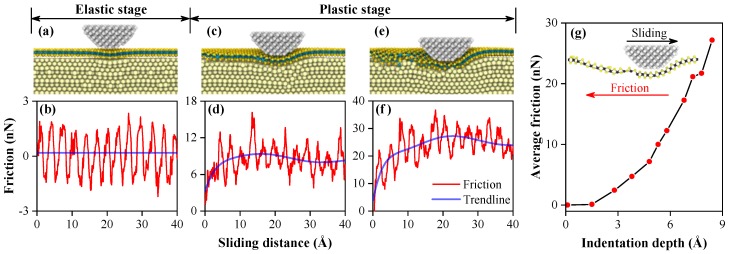
Cutaway view of the model and the friction-distance curves during the sliding process, (**a**,**b**) indentation depth = 1.5 Å at elastic stage; (**c**,**d**) indentation depth = 5.4 Å, at plastic stage; (**e**,**f**) indentation depth = 8.4 Å at the critical loading depth. (**g**) Average friction varies with the indentation depths during the sliding process.

During the indentation process, the results shows that the structural deformation of MoS_2_ affects the anti-pressure performance of monolayer MoS_2_. The atomistic nano-scratch process with an ideal monolayer of MoS_2_ supported by a platinum substrate has been simulated to investigate the friction reduction effect of monolayer MoS_2_ before studying the effects of compressive prestrain on the anti-wear performance of monolayer MoS_2_, as shown in Figure 6. It could be found that the friction force is modulated with the periodicity of the atomic lattice at small indentation depth (the elastic stage). This is consistent with the characteristics of stick slip, as shown in Figure 6a,b. With further increases of indentation depth, the stick slip disappeared gradually, and the friction-distance curve is increasingly irregular, as shown in Figure 6c–f. In order to visually compare the friction reduction effect of monolayer MoS_2_ under various indentation depths, the average friction values have been calculated, as shown in Figure 6g. With increasing indentation depth, the average friction increases correspondingly, which is consistent with a previous study [48]. The results above indicate that the friction reduction effect of monolayer MoS_2_ depends on the deformation degree of monolayer MoS_2_. MoS_2_ offers excellent lubricity at infinitesimal deformation. While the lubrication performance decreases with the increasing deformation. However, the excellent lubricity of monolayer MoS_2_ will disappear once the MoS_2_ layer is ruptured.

**Figure 7:**
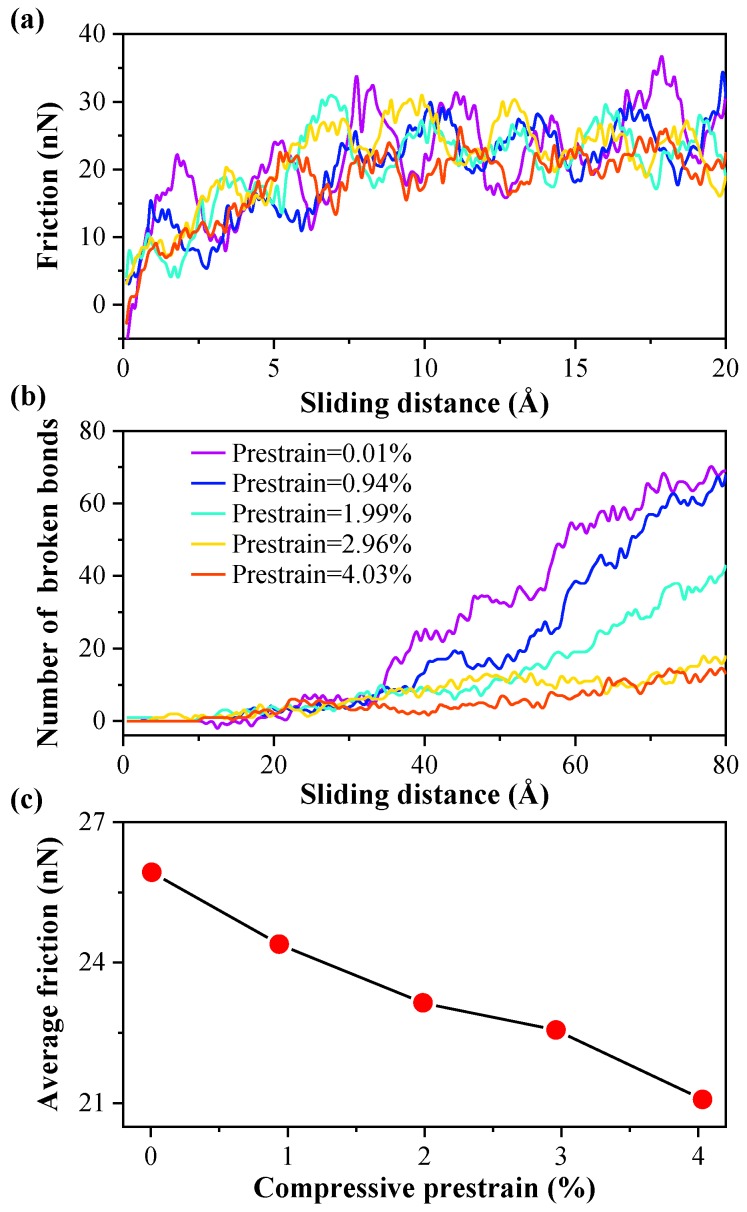
During the sliding process, the friction-distance relation curves (**a**) and the number of broken bonds (**b**) of each model at the critical loading depth with ideal monolayer MoS_2_ (8.4 Å). (**c**) The average friction under various compressive prestrains, obtained by averaging the friction force when the friction is stable.

To compare the anti-wear performance of MoS_2_ with different compressive prestrains, analogous simulations with prestrain models have been performed at the critical loading depth with ideal monolayer MoS_2_. From the Figure 7a, the friction-distance curve of each model loses its cyclic tendency due to stick slip and becomes irregular, which is consistent with the trend at the plastic stage during sliding process. To measure the wear of MoS_2_ in each model, the number of broken bonds is calculated as shown in Figure 7b. During the sliding process, the number of broken bonds in each model is similar at the beginning of the sliding process. It increases in varying degrees with further increases of sliding distance. The growth rate of the number of broken bonds decreases with the increasing in-plane compressive prestrain of monolayer MoS_2_. It could be considered that the breaking and recombining events of Mo–S bonds coexist simultaneously at the critical loading depth. The greater the in-plane compressive prestrain of monolayer MoS_2_ is, the closer the breaking and recombining events of the Mo–S bond are in approaching equilibrium. The monolayer MoS_2_ is more difficult to rupture in large in-plane compressive prestrain during the sliding process because the broken Mo–S bonds readily recombine once the diamond tip slides away.

From Figure 7a, the friction force increases significantly with the increase of the sliding distance at the beginning of the sliding process. In order to accurately evaluate the anti-wear performance of MoS_2_ with different compressive prestrains, the average friction forces are calculated by averaging the friction force when the friction is stable, as shown in Figure 7c. It could be found that the average friction force of each model decreases gradually with increasing in-plane compressive prestrain of monolayer MoS_2_. It is proven that the anti-wear performance is improved under in-plane compressive prestrain condition.

**Figure 8:**
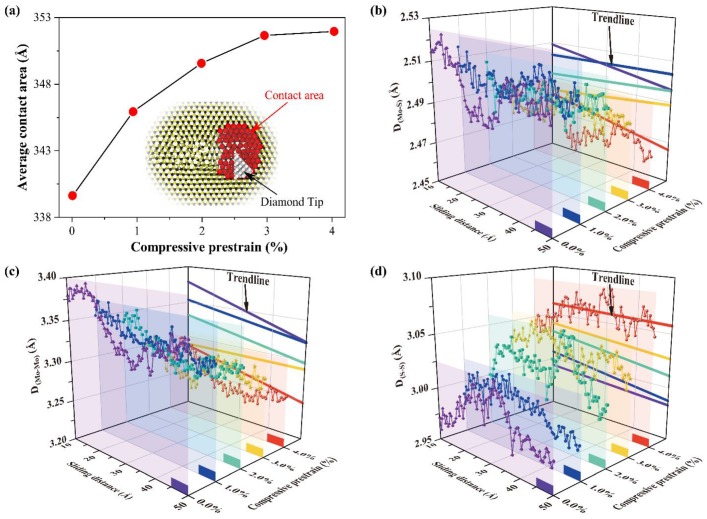
(**a**) The average contact area between the diamond tip and MoS_2_ with different in-plane compressive prestrain, obtained by averaging the contact area during the sliding process; Evolution of D_(Mo–S)_ (**b**), D_(Mo–Mo)_ (**c**), and D_(S–S)_ (**d**) for atoms in the contact area between the diamond tip and MoS_2_, when the friction is stable.

In order to explain why in-plane compressive prestrain is able to enhance the anti-wear performance of the monolayer MoS_2_, the average contact area between the diamond tip and MoS_2_ is calculated, as shown in Figure 8a. When the in-plane compressive prestrain increases, the contact area between the diamond tip and MoS_2_ increases correspondingly. Generally, the diamond tip presents a much harder potential barrier to overcome the increase of the contact area [55], which leads to an increase on the average friction force. However, in this work, the average friction force decreases gradually with the increasing in-plane compressive prestrain of monolayer MoS_2_. A previous study [36] has reported the effect of prestrain on the tribological behavior between MoS_2_ layers by density functional theory (DFT). It is found that the compressive prestrain reduces the friction between MoS_2_ layers due to the enhanced coulombic repulsive interaction. The further research finds that the structural deformations during the sliding process enhance the anti-wear performance of the monolayer MoS_2_, as shown in Figure 8b–d. The trend of D_(Mo–S)_, D_(Mo–Mo)_, and D_(S–S)_ curves show no obvious variation with increasing sliding distance. However, they are modulated by the in-plane compressive prestrain of monolayer MoS_2_. With the increase of the in-plane compressive prestrain, the position of D_(Mo–Mo)_ curve tends to decrease, while the position of D_(S–S)_ curve rises. That is, the degrees of the in-plane tensile deformation and out-of-plane compressive deformation are reduced with the in-plane compressive prestrain increases, resulting in the improvement of the anti-wear performance of the monolayer MoS_2_.

## 4. Conclusions

In conclusion, the anti-pressure and anti-wear performances of monolayer MoS_2_ with the in-plane compressive prestrain have been investigated by means of MD simulations. On ae flat surface of a Pt(111) substrate, the surface roughness of the monolayer MoS_2_ increases slightly with the increasing in-plane compressive prestrain. There are three deformation stages of MoS_2_/Pt substrate during the nano-indentation process, the elastic stage, plastic stage and complete rupture stage, respectively. The monolayer MoS_2_ clearly improves the load bearing capacity of the Pt substrate during the elastic and plastic stages. With increasing in-plane compressive prestrain, the capacities of MoS_2_ for resisting both the in-plane tensile deformation and out-of-plane compressive deformation are improved, resulting in an enhancement of the anti-pressure performance of monolayer MoS_2_. The results show that both capacities to resist the tensile and compressive deformation are improved when the compressive prestrain is less than 2%. The capacity to resist the tensile deformation is responsible for the improvement of the anti-pressure performance when the compressive prestrain increases further.

The friction reduction effect of monolayer MoS_2_ depends on the deformation degree of monolayer MoS_2_. MoS_2_ shows excellent lubrication performance at low indentation depths, while the lubrication property of monolayer MoS_2_ is lost once the MoS_2_ layer is ruptured. The anti-wear performance of monolayer MoS_2_ can be enhanced by increasing in-plane compressive prestrain, owing to the reduction of the in-plane tensile deformation and out-of-plane compressive deformation of the monolayer MoS_2_. These results may provide guidelines for optimizing the anti-pressure and anti-wear performance of monolayer MoS_2_ and other two-dimensional materials which are subjected to in-plane prestrain.

## Figures and Tables

**Table 1:** Prestrain parameters of MoS_2_ along the *x*-direction

Model	Compressive Prestrain	x Size Before Compression (Å)	x Size After Compression (Å)
Ⅰ	0.01%	113.774	113.768
Ⅱ	0.99%	148.539	147.066
Ⅲ	1.99%	135.897	133.192
Ⅳ	2.96%	120.095	116.543
Ⅴ	4.03%	135.897	130.417

**Table 2:** Parameters of LJ potentials used in the simulation

Parameter	C–S	C–Mo	C–Pt	S–Pt	Mo–Pt
*ε* (meV)	13.165	48.962	38.635	177.840	661.41
σ (Å)	3.418	3.009	2.971	2.922	2.513
*R*_cutoff_ (Å)	8.545	7.523	7.428	7.305	6.283

## Supplementary Materials

The following are available online at https://www.mdpi.com/2079-4991/10/2/275/s1, Figure S1: Schematic of two thermostat schemes: (a) Part NVT: the Langevin thermostat is only applied to the MoS_2_ atoms close to the fixed region. (b) All NVT: the Langevin thermostat is applied to all free atoms from the MoS_2_ layer. Part NVT is adopted in this work. For both schemes, the thermosetting regions in the Pt substrate are the same as that in the main manuscript. (c) The force-depth relations of the MoS_2_/Pt substrate during nano-indentation process using Part NVT and All NVT. (d) The friction-distance curves of the MoS_2_/Pt substrate during nano-scratch process using Part NVT and All NVT. Figure S2: Schematic of indenting points (IP) and scanning lines (SL) on the MoS_2_. Each scanning line passes through a corresponding indenting point, which is indicated by red cross.
