# Supplementary material for: Effect of Compressive Prestrain on the Anti-Pressure and Anti-Wear Performance of Monolayer MoS2: A Molecular Dynamics Study
Source: Nanomaterials (Basel). 2020 Feb 6;10(2):275. doi: 10.3390/nano10020275 (PMC7075122; doi:10.3390/nano10020275)
Supplement: Supplementary file 1 [file nanomaterials-10-00275-s001.pdf]

nanomaterials

MDPI

Supplementary

Effect of Compressive Prestrain on the Anti-Pressure and Anti-Wear Performance of Monolayer MoS_2: A Molecular Dynamics Study

Ning Kong [1,*], Boyu Wei [1], Yuan Zhuang [2], Jie Zhang [1], Hongbo Li [1], and Bo Wang [2]

[1] School of Mechanical Engineering, University of Science and Technology Beijing, Beijing 100083, China; g20188554@xs.ustb.edu.cn (B.W.); ZhangJie@ustb.edu.cn (J.Z.); lihongbo@ustb.edu.cn (H.L.)

[2] Beijing Institute of Spacecraft System Engineering, Beijing 100094, China; zyuan8707@163.com (Y.Z.); wb861226@126.com (B.W.)

* Correspondence: kongning@ustb.edu.cn

Received: 02 January 2020; Accepted: 03 February 2020; Published: 06 February 2020

1. Thermosetting Scheme

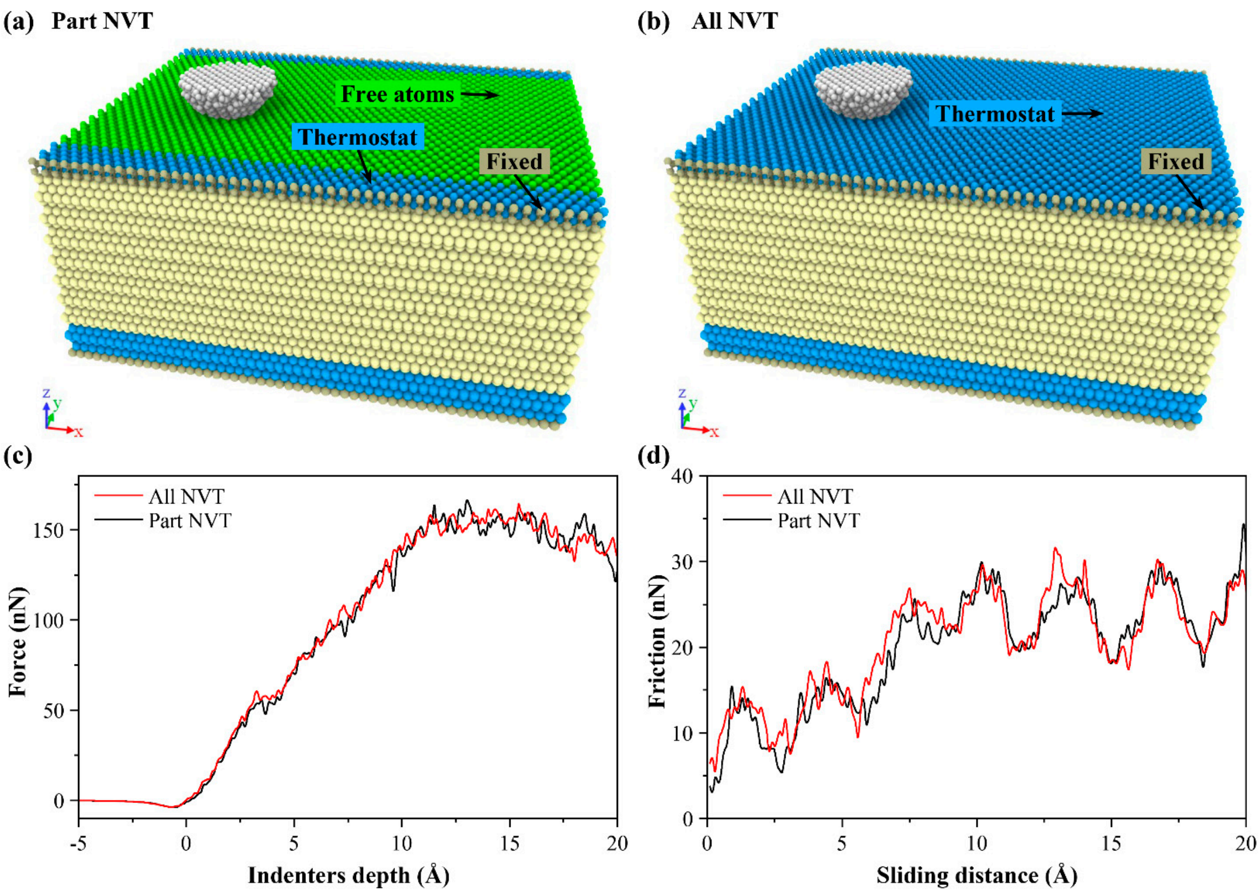

Figure S1. Schematic of two thermostat schemes: (**a**) Part NVT: the Langevin thermostat is only applied to the MoS_2 atoms close to the fixed region. (**b**) All NVT: the Langevin thermostat is applied to all free atoms from the MoS_2 layer. Part NVT is adopted in this work. For both schemes, the thermosetting regions in the Pt substrate are the same as that in the main manuscript. (**c**) The force-depth relations of the MoS_2/Pt substrate during nano-indentation process using Part NVT and All

NVT. (**d**) The friction-distance curves of the MoS_2/Pt substrate during nano-scratch process using Part NVT and All NVT.

2. Indenting Points and Scanning Lines

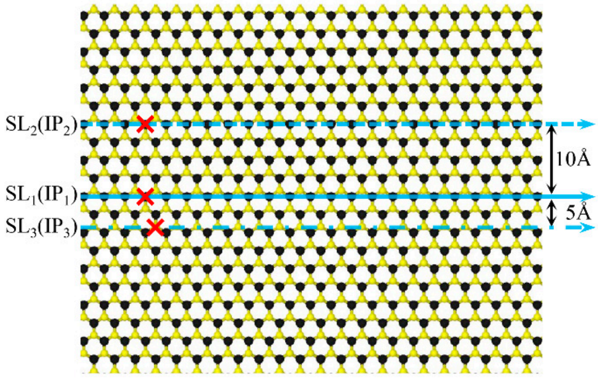

Figure S2. Schematic of indenting points (IP) and scanning lines (SL) on the MoS_2. Each scanning line passes through a corresponding indenting point, which is indicated by red cross.
